# State-Level Policy Actions to Support Family Caregivers

**DOI:** 10.1093/geroni/igab046.245

**Published:** 2021-12-17

**Authors:** Molly Evans

**Affiliations:** Commonwealth of Massachusetts, Boston, Massachusetts, United States

## Abstract

The stresses created by the growing need for family caregivers have failed to prompt federal policy action; in its absence, states are stepping up. This review of state policies that support employment among family caregivers found six main categories of legislative action: paid leave; expanding federally mandated unpaid leave; paid sick time; unemployment insurance for job loss attributable to caregiving duties; establishing family caregivers as a protected classification in employment discrimination; and flexible or alternative work schedules. Despite the demand for policies that support and empower working caregivers, a minority of states have passed such legislation; to date, 9 states have implemented paid family leave; 14 have implemented mandatory sick leave legislation; and 14 have expanded FMLA. This study discusses state-level policy actions, reviews the status and importance of these policies, and finds that despite gaps in caregiver support legislation at the state level, there is significant and promising momentum.

